# Current Practices and Challenges in the Diagnosis and Management of PKU in Latin America: A Multicenter Survey

**DOI:** 10.3390/nu13082566

**Published:** 2021-07-27

**Authors:** Soraia Poloni, Bruna Bento dos Santos, Ana Chiesa, Norma Specola, Marcela Pereyra, Manuel Saborío-Rocafort, María Florencia Salazar, María Jesús Leal-Witt, Gabriela Castro, Felipe Peñaloza, Sunling Palma Wong, Ramsés Badilla Porras, Lourdes Ortiz Paranza, Marta Cristina Sanabria, Marcela Vela Amieva, Marco Morales, Amanda Rocío Caro Naranjo, Antonieta Mahfoud, Ana Rosa Colmenares, Aida Lemes, José Fernando Sotillo-Lindo, Ceila Perez, Laritza Martínez Rey, Georgina María Zayas Torriente, Lilia Farret Refosco, Ida Vanessa Doederlein Schwartz, Veronica Cornejo

**Affiliations:** 1 Hospital de Clínicas de Porto Alegre, Porto Alegre 90035-903, Brazil; brunabentods@gmail.com (B.B.d.S.); lrefosco@hcpa.edu.br (L.F.R.); idadschwartz@gmail.com (I.V.D.S.); 2 Genetics and Molecular Biology, Universidade Federal do Rio Grande do Sul, Porto Alegre 91501-970, Brazil; 3 Centro de Investigaciones Endocrinologicas DR Cesar Bergadá, CEDIE-CONICET-Fundación de Endocrinologia Infantil-Division de Endocrinologia Hospital de Niños R Gutierrez, Gallo 1330, Buenos Aires C1425EFD, Argentina; achiesa@cedie.org.ar; 4 Unidad de Metabolismo, Hospital de Niños de La Plata, La Plata B1904, Argentina; normaspecola@gmail.com; 5 Servicio de Crecimiento y Desarrollo del Hospital Pediátrico Dr. H. Notti, 2603, Mendoza M5519, Argentina; marcela_pereyra@hotmail.com; 6 Hospital Nacional de Niños, Caja Costarricense de Seguro Social & Sistema de Estudios de Posgrado, Universidad de Costa Rica, San José 11501, Costa Rica; MANUEL.SABORIO@ucr.ac.cr; 7 Instituto de Nutrición y Tecnología de los Alimentos (INTA) Universidad de Chile, Santiago de Chile 1058, Chile; mfsalazar@inta.uchile.cl (M.F.S.); mj.leal@inta.uchile.cl (M.J.L.-W.); gcastro@inta.uchile.cl (G.C.); felipe.penaloza@inta.uchile.cl (F.P.); vcornejo@inta.uchile.cl (V.C.); 8 Programa Nacional de Tamizaje, Hospital Nacional de Niños, San José 267-1005, Costa Rica; spalmaw@tamizajecr.com; 9 FCCMG Servicio de Genética Médica y Metabolismo, Hospital Nacional de Niños, San José 267-1005, Costa Rica; rbadillap@tamizajecr.com; 10 Ministerio de Salud Pùblica y Bienestar Social, Asunciòn 1735, Paraguay; lrortizp@yahoo.es; 11 Pediatric Department and Department of the Hospital de Clínicas, Universidad Nacional de Asunciòn, Asunción 1102, Paraguay; marta.sanabria@gmail.com; 12 Laboratorio de Errores Innatos del Metabolismo y Tamiz-Instituto Nacional de Pediatría, Ciudad de México 04530, Mexico; dravelaamieva@yahoo.com; 13 Hospital Rebagliati, Lima 15072, Peru; moralesmarco2004@yahoo.com; 14 Instituto de Errores Innatos del Metabolismo de la Pontificia Universidad Javeriana, Bogota 110231, Colombia; amanda.caro@javeriana.edu.co; 15 Instituto de Estudios Avanzados, Caracas 17606, Venezuela; amahfoud@gmail.com; 16 Hospital Clinica Caracas-Materno Infantil de Caricuao, Caracas 1000, Venezuela; anarosacol@hotmail.com; 17 Instituto de la Seguridad Social, Montevideo 11000, Uruguay; lemesa@adinet.com.uy; 18 Hospital de especialidades Pediátricas “Omar Torrijos Herrera”, Ciudad de Panamá 07136, Panama; jfsotillotuto_2000@hotmail.com; 19 Robert Reid Cabral Children’s Hospital, Santo Domingo 10101, Dominican Republic; cepef@hotmail.com; 20 Centro Nacional de Genética Médica, La Habana 11300, Cuba; laritzam@infomed.sld.cu; 21 Centro de Nutrición e Higiene de los Alimentos del Instituto Nacional de Higiene, Epidemiología y Microbiología (INHEM), La Habana 10300, Cuba; georgina.zayas@infomed.sld.cu

**Keywords:** phenylketonuria, PKU, low-protein diet, newborn screening

## Abstract

This study aimed to describe the current practices in the diagnosis and dietary management of phenylketonuria (PKU) in Latin America, as well as the main barriers to treatment. We developed a 44-item online survey aimed at health professionals. After a pilot test, the final version was sent to 25 practitioners working with inborn errors of metabolism (IEM) in 14 countries. Our results include 22 centers in 13 countries. Most countries (12/13) screened newborns for PKU. Phenylalanine (Phe) targets at different ages were very heterogeneous among centers, with greater consistency at the 0–1 year age group (14/22 sought 120–240 µmol/L) and the lowest at >12 years (10 targets reported). Most countries had only unflavored powdered amino acid substitutes (10/13) and did not have low-protein foods (8/13). Only 3/13 countries had regional databases of the Phe content of foods, and only 4/22 centers had nutrient analysis software. The perceived obstacles to treatment were: low purchasing power (62%), limited/insufficient availability of low-protein foods (60%), poor adherence, and lack of technical resources to manage the diet (50% each). We observed a heterogeneous scenario in the dietary management of PKU, and most countries experienced a lack of dietary resources for both patients and health professionals.

## 1. Introduction

Latin America comprises 20 countries and has an ethnically diverse population of over 650 million people. With a complex political and economic background, these countries face many challenges in the diagnosis and care of patients with inborn errors of metabolism (IEM), such as phenylketonuria (PKU, OMIM #261600). The success of early diagnosis and dietary treatment of PKU has been well described since the 1960s. Since then, and until the mid-1970s, most developed countries have initiated national newborn screening (NBS) programs for PKU [1]. In Latin America, the first organized NBS programs were only started in 1986 in Cuba, followed by Costa Rica (1990), and Chile (1992) [2]. At present, 16 countries have national or regional NBS programs, but only 6 have coverage ≥90% [3].

Similarly to NBS programs, PKU management faces many challenges in Latin America. Despite significant health system reforms in the 1980s, inequality and impaired access to health care remains a major problem in the region [4]. A recent report by the Organization for Economic Co-operation and Development (OECD) showed that government and compulsory health insurance represented only 54.3% of the current expenditure on health in Latin America, with 34% of all health spending being paid out-of-pocket. Nearly 8% of the Latin American population spends more than 10% of their household consumption or income on health care services. Latin American countries also have a much lower availability of medical technologies and health professionals when compared to other countries [5].

Treatment of PKU inflicts a substantial time and cost burden on patients and their families [6], and this can be a significant obstacle to encouraging patients to remain on a restricted diet [7]. Moreover, a trained health care team is needed to manage the extremely restrictive diet and to educate patients and families, and frequent laboratory tests are required to guide the treatment. The current situation of PKU diagnosis and management in Latin America is unknown, since only sparse and country-based reports have been published [8,9,10,11,12]. Therefore, the aim of this study was to map the current practices in the diagnosis and dietary management of PKU in Latin America, as well as the main barriers to treatment perceived by health care providers.

## 2. Materials and Methods

### 2.1. Study Design

A questionnaire containing 44 questions on the diagnosis and management of PKU was developed by a team of experts from Brazil (S.P., B.B.S., I.V.D.S., and L.F.R.) and Chile (M.J.L., F.S., G.C., and V.C.). These were experienced metabolic dietitians and geneticists, all co-authors of this paper. The survey had multiple choice and short answer questions and was aimed at health care professionals following patients with PKU. Five main issues were addressed: features and professional training of the health care team, newborn screening, treatment goals and dietary practices, availability of alternative treatments, and perceived barriers to treatment.

After the first Portuguese and Spanish versions of the questionnaire were finished, a pilot study was performed with 6 PKU experts (3 Portuguese and 3 Spanish speakers) to identify possible flaws or misinterpretations of the questions. Only minor adaptations were made, and the final version was then shared on an online platform.

To disseminate the survey, practitioners of IEM were searched for (through public archives of the Sociedad Latinoamericana de Errores Innatos del Metabolismo y Pesquisa Neonatal in all Latin American countries, and were found in 14 of them. The coordinator team designated 1 responsible person in each country to distribute the survey to other centers nationally. The aim was to distribute the survey to as many centers as possible in each country. The only exception was Brazil, the largest country with the most PKU treatment centers (>20); to avoid overrepresentation, we chose 1 center from each region of the country. The invitation and distribution of the survey was performed by e-mail from July to November 2020. The final version of the questionnaire is available by request.

### 2.2. Ethical Aspects

The study was approved by the Research Ethics Committee of Hospital de Clínicas de Porto Alegre, Brazil (CAAE 36401120.6.0000.5327), and the survey was initiated only after the participants agreed with the online informed consent form.

## 3. Results

### 3.1. Participants

Out of the 14 contacted country representatives, 13 were following patients with PKU, and all of them agreed to participate in the study. In total, 22 treatment centers were enrolled from the following countries: Brazil (*n* = 5), Argentina (*n* = 4), Colombia (*n* = 2), Venezuela (*n* = 2), Costa Rica (*n* = 1), Chile (*n* = 1), Mexico (*n* = 1), Paraguay (*n* = 1), Peru (*n* = 1), Dominican Republic (*n* = 1), Panama (*n* = 1), Uruguay (*n* = 1), and Cuba (*n* = 1).

The respondents were mostly female (91%), were aged ≥45 years (61%), and had worked with PKU for over 10 years (70%). Physicians represented 59% of the respondents, with the remaining respondents being dietitians. Regarding professional training, 45% (*n* = 10/22) stated that they had a specialization course in the field, 41% (*n* = 9/22) had only short-term courses, and 9% (*n* = 2) had no formal training. The number of patients with PKU who were followed up by the professionals varied considerably: 18.2% (*n* = 4/22) had <10 patients, 18.2% (*n* = 4/22) had 10–25 patients, 18.2% (*n* = 4/22) had 26–50 patients, 13.6% (*n* = 3/22) had 51–75 patients, and 32% (*n* = 7/22) had >75 patients.

### 3.2. Newborn Screening and Phenylalanine (Phe) Monitoring

**Figure 1:**
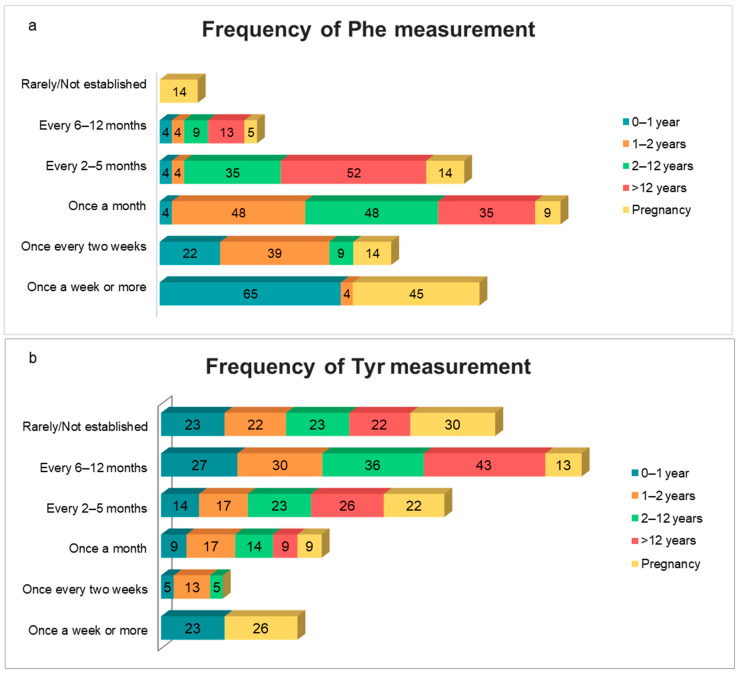
Frequencies of blood phenylalanine (Phe, **a**) and tyrosine (Tyr, **b**) monitoring for each age group as adopted by the Latin American centers included in the study (*n* = 22). Numbers within columns represent relative percentages.

Regarding NBS, all countries but one (Dominican Republic) had a national NBS program, the most recent one being in Colombia (2019). When inquired on the Phe cutoff level used to start dietary treatment, 13/22 centers (59%) responded ≥360 µmol/L (≥6 mg/dL), 5/22 (23%) responded ≥600 µmol/L (≥10 mg/dL), 2/22 (9%) responded <360 µmol/L (<6 mg/dL), and 1 (4.5%) responded ≥480 µmol/L (≥8 mg/dL). In most centers (19/22), blood Phe was measured in dried blood spots. The most used method to analyze blood Phe was the fluorometric assay (12/22), followed by tandem mass spectrometry (5/22). Figure 1 shows the recommended frequency of Phe and tyrosine (Tyr) monitoring in the studied centers.

### 3.3. Treatment Targets and Dietary Practices

**Figure 2:**
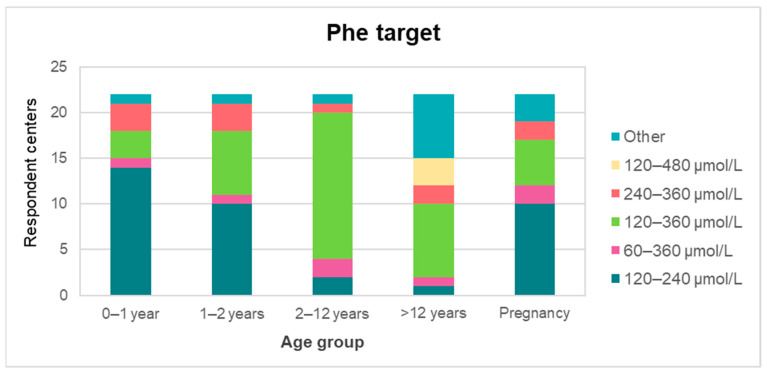
Target Phe levels during treatment in different age groups, as adopted by the studied centers (*n* = 22). Phe: phenylalanine.

**Figure 3:**
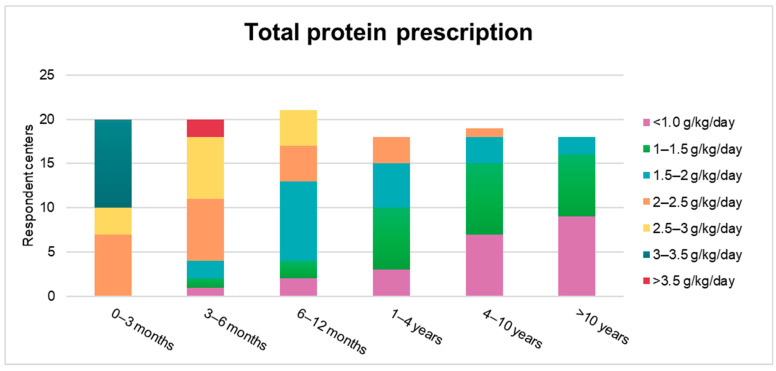
Total protein (natural + protein substitute) prescriptions, in different age groups, in the studied centers (*n* = 22) Some centers did not fully answer this question; therefore, the sample size varies in different age groups.

Figure 2 shows Phe target levels at different ages in the studied centers. Dietary guidance was most frequently performed through the simplified method of high/medium/low Phe content of foods (10/22), followed by individualized meal plans (8/22), and protein counting (3/22). A 24 h dietary recall (or similar) was performed at every appointment in most centers (17/22). Total protein prescriptions are described in Figure 3.

All but two respondents reported that the maintenance of partial breastfeeding was encouraged in classical PKU patients. Most respondents (80%) said that they instructed mothers to offer the protein substitute right before breastfeeding to control Phe intake.

Regarding nutritional monitoring, all centers reported weight and height measurements at every appointment, and 19/22 always assessed head circumference. The evaluation of body composition was less frequent; 12/22 (54%) did not assess skinfolds and none performed bioelectrical impedance analyses on a regular basis. The blood tests that were performed at least once a year were: a complete blood count (95%), fasting glucose (91%), total protein (91%), creatinine (91%), urea (77%), lipoproteins and triglycerides (77%), albumin (73%), vitamins B12 and D (60%), and ferritin (60%). A complete amino acid profile was requested in 10/22 centers (45%), and only 1 center evaluated essential fatty acids on a regular basis. Bone densitometry was routinely performed in 11/22 centers (50%).

### 3.4. Nutritional Resources

Out of the 13 included countries, 9 reported having national guidelines for PKU management, and 12/22 (54%) centers had local management protocols. The theoretical background most commonly used by the respondents was: international guidelines (61%), scientific papers (56%), and national guidelines (48%). Regarding dietary resources, only 18% of the centers (4/22) reported having an adequate regional database of the Phe content of foods, and 33% stated that only an incomplete database was available. The remaining (49%) centers utilized a variety of international databases or considered only the protein content of foods for guiding the diet. Food recalls were usually calculated manually (48%) or through a customized spreadsheet developed by the center (3%). Only 3/22 centers reported having specialized nutrition software.

Except for one country, none of the participant countries had the Phe content available on food labels. Regarding protein substitutes, 11/13 countries had only unflavored powdered amino acid formulas; only 1 country (Argentina) had several options, such as gels and tablets, available. In 10/13 countries, the protein substitute was fully subsidized by the government. Specific low-protein foods for PKU were not available in 8/13 countries; even when these were available, 58% of the centers stated that they were not affordable. These products were subsidized by the government in only 2/13 countries.

### 3.5. Alternative Treatments and Challenges

Six countries had no alternative treatments available. Among those that had them, sapropterin (BH4) was the most frequent (six countries—Argentina, Brazil, Costa Rica, Dominican Republic and Mexico; approximately 60 patients in total); large neutral amino acids (LNAA) were available in two countries (Argentina and Peru), and glycomacropeptide (GMP) was available in one country (Argentina). Argentina was the only country that had all three options available, also with the most patients using them (>20 patients on BH4 and GMP and nearly 10 patients on LNAA).

**Figure 4:**
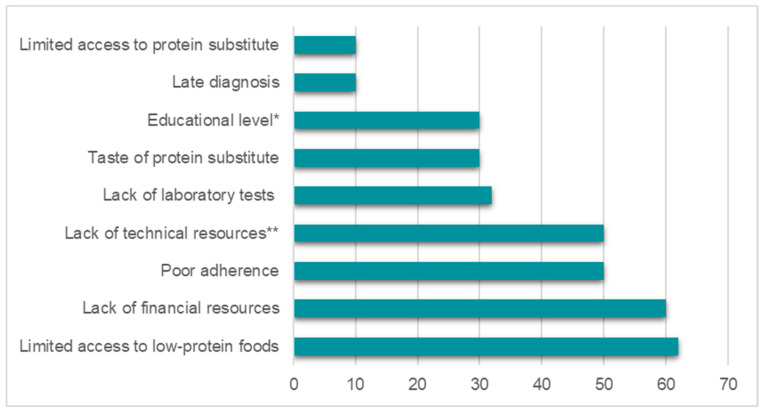
Barriers to treatment most commonly perceived by the respondents (*n* = 22). Values represent the median scores assigned by the respondents. * Educational level of patients and caregivers; ** Technical resources required or desirable to manage the diet, such as a local database of the Phe content of foods and specialized nutrition software.

Participants were asked to provide a score from 0 to 100 on how much they believed each category had contributed to hampering therapeutic success. Median scores are depicted in Figure 4. In addition to the aspects shown in Figure 4, other cited barriers to treatment were: low accessibility due to geographic location, limited access to alternative treatments, high cost of treatments, and long periods of time for samples to arrive at the laboratory.

## 4. Discussion

This study reports a broad and unprecedented characterization of the current state of diagnosis and management of PKU across Latin America. Data on NBS, laboratory tests, professional training, treatment targets, dietary practices, and resources, among other aspects, were compiled from 13 different countries and 22 treatment centers. These countries represent 87% of the Latin American population. Respondents were physicians and dietitians, most of whom were experienced in PKU treatments and were following a variable amount of PKU patients of all ages.

NBS for PKU began mostly after the 1990s in Latin America, nearly 30 years after the USA and some European countries had initiated their screening programs [1,2]. Nevertheless, most Latin American countries currently have wide-coverage national NBS programs for PKU and multidisciplinary reference centers for the follow-up of these patients, as shown in our study. Although the need for NBS and early treatment of PKU was generally agreed upon, other practices were not. Whereas both American and European guidelines [13,14] recommend that treatment should be started when Phe levels are ≥360 μmol/L, 35% (*n* = 8) of the centers in our sample employed different cutoffs, with most of them (*n* = 6) using higher levels. Higher cutoffs could miss mild PKU patients and raise concern due to the detrimental effect of high Phe levels in early life. A meta-analysis showed that each 100 μmol/L increase in Phe in early life predicted a 1.3- to 3.9-point decrease in intelligence quotient (IQ) over a Phe range of 394 to 750 μmol/L [15]. However, the exact cutoff at which treatment should begin is still debatable. There is a consensus that individuals with Phe levels >600 μmol/L should be treated, but the evidence regarding the initiation of treatment with blood Phe concentrations between 360 and 600 μmol/L is inconsistent. Given the risk of neurocognitive consequences, most guidelines recommend initiating treatment when blood Phe concentrations are >360 μmol/L [8,13,14].

The frequency of Phe and Tyr monitoring was highly heterogeneous among centers. The highest agreement (65%) found was in respect to measuring Phe once a week or more in infants younger than 1 year of age (Figure 1), which is in line with both American and European recommendations [13,14,16]. An even greater disagreement was observed for Tyr measurements, in all age groups. This probably reflects the lower availability of Tyr analyses in several centers: more than 20% of them rarely or never measured Tyr, regardless of the patient’s age group or condition. Tyr monitoring is critical in PKU, since this amino acid cannot be synthetized properly due to the metabolic blockage, and a decreased availability of Tyr in the brain likely contributes to the cognitive impairment found in untreated patients [17]. American Genetic Metabolic Dietitians International (GMDI) guidelines recommend that Tyr measurements be performed as frequently as Phe measurements [16].

A similar heterogeneity was observed for Phe target values throughout life (Figure 2). The highest agreement (69%) was for children aged 2–12 years, where the Phe target was 120–360 μmol/L; this was in agreement with both international guidelines [13,14]. For infants younger than 1 year of age, most (61%) centers aimed for Phe levels to be between 120 and 240 μmol/L, a goal that differed from the American and European guidelines, which recommend a Phe target of 120–360 μmol/L [13,14]. Chilean guidelines support the 120–240 μmol/L target at this age, since in this period many factors interfere with the Phe level, such as growth, teething, infections, and frequent vaccinations [8]. The age group with the highest heterogeneity was >12 years, with 11 different targets reported. Eight (35%) centers agreed with the American target (120–360 μmol/L), and two (9%) agreed with the European values (120–600 μmol/L). A trend towards more restrictive targets was observed in all age groups and in pregnant patients. However, the theoretical basis for some of the reported targets was not clear; in some cases they were unique and diverged within the same country, even when national guidelines were available.

Greater consistency was found in dietary practices. The simplified method was the most frequently used approach to manage dietary intake (in 48% of the centers). The simplified diet approach has been shown to be easier to follow, encourages healthy food choices, and can improve the quality of life and adherence of patients with PKU [18,19]. Breastfeeding was encouraged in most centers, reflecting its clear evidence-based benefits in PKU [20,21]. Regarding nutritional monitoring, basic measurements such as weight, height, head circumference, and food recalls were performed in most centers at all appointments, meeting international recommendations [13,14]. Blood tests for nutritional monitoring were usually performed on a regular basis. It is noteworthy, however, that pivotal examinations such as amino acid profiles and bone densitometry were not regularly performed in most centers (56%). These assessments are required in the follow-up of patients with PKU who are being treated, since they are at risk of amino acid deficiencies and osteopenia [13,14,17,22]. A likely explanation for this is that these two technologies are less available due to their high costs and need for specialized facilities. There are substantial differences in the availability of technologies across Latin American countries [5]. The total protein prescriptions showed some heterogeneity among centers (Figure 3). However, the well-established recommendation that a higher protein intake is necessary for patients with PKU [23] has been mostly followed.

Nutritional resources to support patients, families, and professionals were scarce. Although most countries had national guidelines, most respondents reached for international guidelines (61%) as theoretical background. This might be due to outdated or incomplete local guidelines. While most professionals used Phe intake for managing the diet, most of them (78%) did not have a suitable regional database of the Phe content of foods and had to rely on international databases. However, nutrient contents of foods can vary due to environmental factors, production, and processing, and might differ between countries [24]. Health professionals also face difficulties calculating the diets: only four (17%) centers had specialized nutrition software.

For patients, unflavored powdered amino acid formulas were the only protein substitute available in most countries. Specific low-protein foods for PKU were unavailable in 61% of the countries. Even when available, they were usually not affordable, since these were rarely subsidized by the government. Specially designed low-protein products are important for satiety and diet variety [25], and were also proven valuable in improving metabolic control and growth in patients with PKU [26]. However, they inflict a significant financial burden to the PKU diet: in an American study, low-protein foods represented the highest annual out-of-pocket costs (child = US$1651.00; adult = US$967.00) when compared to other categories of care [6]. Considering the gross national income per capita in 2019 [27], this would be equivalent to 20% of the income of a Latin American citizen. The average expenditure on food of a Brazilian citizen, for instance, is USD 866.00 per year. Therefore, it is completely unreasonable to expect that Latin American patients with PKU would be able to afford low-protein foods without subsidy.

Alternative therapies are also a reality for a few in Latin America. BH4 was the most common therapy, available in 7/13 countries. However, even when approved, this therapy was only used in a few patients. LNAA and GMP were even rarer, despite several products being available in Europe and the USA for years [28]. Alternative treatments are highly relevant in PKU since most patients struggle to follow the restrictive diet and to take the protein substitute [29,30]. As a consequence of suboptimal metabolic control and restrictive dietary management, psychiatric illness is common in adult PKU patients. The advent of new treatments that do not require such a restricted diet might improve metabolic control, mental health, and cognitive functioning in these patients [31].

Finally, we asked the respondents to score the topics they considered the greatest barriers to the adequate treatment of PKU in their realities. The answers largely reflected the major gaps found throughout the study: lack of nutritional resources for patients and professionals and the high cost of therapies. One of the highest assigned scores was for “poor adherence”, which may also be an outcome of the difficulties mentioned above. Another barrier cited by the respondents was low accessibility due to geographic location. In Latin America, most of the sophisticated technologies that are required for the follow-up of patients with IEM are geographically concentrated in larger and wealthier urban areas, contributing to health inequalities in this population [5].

## 5. Conclusions

In conclusion, here we have reported the first compilation of the status of PKU care in Latin America. Despite most countries having national NBS programs and guidelines, we found a highly heterogeneous scenario considering practices across countries and even within the same country. The struggles, however, were similar. Most countries experienced a lack of resources for both patients and health care professionals, which may be impairing treatment outcomes. Together, these results indicate an urgent need for a comprehensive Latin American guideline that must be able to integrate the latest evidence-based recommendations with the challenges and possibilities faced by Latin American countries.

## Data Availability

Data available on request due to restrictions eg privacy or ethical. The data presented in this study are available on request from the corresponding author. The data are not publicly available due to containing information that could compromise the privacy of research participants.
